# Multilevel Exploration of Shared Genetic Architecture Between Primary Biliary Cholangitis and Four Autoimmune Diseases

**DOI:** 10.2174/0118715303446735260429075429

**Published:** 2026-05-17

**Authors:** Chao Shang, Yuanyuan Bai, Yong Zhao

**Affiliations:** 1 Department of General Surgery, The Affiliated Jiangning Hospital of Nanjing Medical University, Nanjing, China

**Keywords:** Primary biliary cholangitis, autoimmune diseases, genetic risk loci, genetic structure, comorbidity analysis, genome-wide analysis

## Abstract

**Introduction:**

Primary Biliary Cholangitis (PBC) frequently coexists with various autoimmune diseases, such as Multiple Sclerosis (MS), Psoriasis (PS), Rheumatoid Arthritis (RA), and Sjögren’s Syndrome (SS). Understanding the genetic associations between these diseases is crucial for providing deeper insights into their shared pathogenic mechanisms and comorbidity patterns.

**Methods:**

This study utilized genome-wide association study summary data of PBC and four autoimmune diseases (MS, PS, RA, and SS). A multi-stage analytical pipeline was employed to systematically investigate the genetic associations between the diseases. The analytical approach consisted of three stages: first, linkage disequilibrium score regression and high-definition likelihood methods were applied to estimate overall genetic correlations between the diseases; second, local genetic correlation analysis was conducted to pinpoint genetic signals in specific chromosomal regions; third, conditional/conjunctional false discovery rate (cond/conjFDR) algorithms were used to quantitatively assess genetic overlap and identify shared susceptibility loci.

**Results:**

Genome-wide analysis revealed significant genetic associations between PBC and the four autoimmune diseases (MS, PS, RA, and SS). Regional analysis showed local genetic correlations across various chromosomal segments. cond/conjFDR analysis confirmed genetic intersections among the diseases and identified several critical genetic polymorphic loci that influence disease susceptibility.

**Discussion:**

This study comprehensively delineates the shared genetic architecture underlying PBC and four autoimmune diseases through integrative analyses of multiple genome-wide approaches. The results highlight strong genetic correlations, particularly between PBC and MS, PS, RA, and SS, and identify key shared susceptibility genes, including CLEC16A, CD58, CD86, STAT4, IRF5, TYK2, and TNFAIP3, which collectively mediate immune dysregulation through autophagy, cytokine signaling, and NF-κB pathways. These findings not only extend current understanding of the molecular mechanisms driving autoimmune comorbidity but also provide potential genetic targets for future functional validation and therapeutic exploration.

**Conclusion:**

This study provides comprehensive genomic evidence for the genetic connections between PBC and the four autoimmune diseases (MS, PS, RA, and SS), offering valuable insights into the shared pathological mechanisms underlying their comorbidities.

## 1 INTRODUCTION

Primary Biliary Cholangitis (PBC) is a chronic, progressive autoimmune liver disorder that primarily affects intrahepatic small bile ducts, resulting in cholestasis, hepatic fibrosis, and eventually advancing to cirrhosis and liver failure [1]. In recent years, the incidence of PBC has exhibited an upward trend. It is estimated that the global incidence and prevalence of PBC are 1.76 and 14.6 cases per 100,000 individuals, respectively [2]. The clinical manifestations of PBC display considerable heterogeneity, with most patients being asymptomatic during the early stages of the disease, often identified through elevated levels of alkaline phosphatase and γ-glutamyltransferase during routine physical examinations. As the disease progresses, patients may experience characteristic symptoms such as fatigue and pruritus, while more severe cases may present with jaundice, hepatosplenomegaly, ascites, and esophageal variceal bleeding [3]. PBC patients typically exhibit various extrahepatic manifestations, particularly certain autoimmune disorders, including Multiple Sclerosis (MS) [4], Psoriasis (PS) [5], Rheumatoid Arthritis (RA) [6], and Sjögren’s Syndrome (SS) [7]. Prior research on the comorbidity of these diseases has predominantly relied on traditional observational study designs. However, such approaches are limited by methodological issues, including inadequate control of confounding factors, sample selection bias, and missing follow-up data, all of which may undermine the reliability of research findings [8]. Genome-wide Association Studies (GWAS), as a contemporary research strategy, offer a novel theoretical framework and technical support for investigating genetic associations between diseases. This molecular genetic approach can effectively overcome the inherent limitations of traditional epidemiological studies and provide comprehensive insights into the molecular mechanisms underlying disease from a genetic perspective [9].

Genetic association analysis is grounded in rigorous statistical methodological frameworks. Recent studies have utilized comprehensive multivariate analytical strategies to successfully elucidate genetic association patterns in various complex diseases: potential candidate gene loci for Alzheimer’s disease and related cognitive disorders have been identified [10], extensive polygenic sharing between inflammatory bowel disease and venous thromboembolism events has been confirmed [11], and the genetic foundation for the comorbidity between autism spectrum disorders and cognitive traits has been uncovered [12]. Building on these methodological advancements, this study was designed to systematically examine the genetic association characteristics between PBC and four autoimmune diseases (MS, PS, RA, SS).

This study was conducted from two perspectives: evaluating the degree of genetic association between the two diseases and identifying shared genetic variant loci. Methodologically, several statistical genetic tools were employed: linkage disequilibrium score regression (LDSC) [13] and High-Definition Likelihood (HDL) [14] were used to analyze genome-wide genetic correlations, while local Analysis Of Variant Association (LAVA) [15] was utilized for detailed analysis of specific regions. To ensure reliable results, the conditional/conjunctional false discovery rate (cond/conjFDR) method [16] was used to identify significant genetic overlap loci. These research findings will facilitate a better understanding of genetic connections between diseases and provide new scientific evidence for precision treatment.

## 2 METHODS AND MATERIALS

### 2.1 Study Design and Type of Study

This study was designed as a genetic association study using GWAS summary data. It primarily aimed to investigate the shared genetic architecture between PBC and four autoimmune diseases (MS, PS, RA, SS).

### 2.2 Data Collection Methods

The selection of research data was guided by multiple evaluative criteria, encompassing essential indicators such as sample size, genetic variant coverage, and the timeliness of data updates. GWAS data for PBC were derived from an international genome-wide meta-analysis performed by Cordell *et al.* [17], while genomic association information for the four autoimmune diseases (MS, PS, RA, SS) was procured from the FinnGen database, version R12 (https://r12.finngen.fi/) [18]. Basic information for each study cohort is provided in Table **1**.

To ensure the reliability of the data analysis, strict quality control measures were implemented. Genetic variants were included only if they met the following criteria: (1) the 1000 Genomes Project Phase 3 served as the reference panel; (2) the minor allele frequency in European populations was above 0.01; and (3) only biallelic variants were considered. The selected variants were annotated based on their positions in the human reference genome (hg19/GRCh37), with variants missing rsID identifiers or showing inconsistent annotations excluded [19]. Given the genetic variation across ethnic groups, this study focused exclusively on data from European populations. The comprehensive research design workflow is shown in Fig. (**1**).

To ensure the integrity of the data analysis, stringent quality control standards were implemented. Genetic variants were included in the analysis only if they satisfied the following three criteria: (1) the 1000 Genomes Project Phase 3 was used as the reference panel; (2) the minor allele frequency was greater than 0.01 in European populations; and (3) only biallelic variants were included. The selected variants were annotated with their genomic positions according to the human reference genome (hg19/GRCh37), and variants lacking rsID identifiers or exhibiting conflicting rsID annotations were excluded. Given the genetic diversity among ethnic groups, this study specifically focused on data from European populations. The specific research design workflow is depicted in Fig. (**1**).

### 2.3 Statistical Methods Used for Analysis

#### 2.3.1 Global Genetic Correlation Analyses

In applying LDSC to analyze the global genetic correlation between PBC and four autoimmune diseases (MS, PS, RA, SS), the fundamental approach relies on GWAS summary statistics for each disease, estimating their shared genetic basis through cross-trait Linkage Disequilibrium (LD) score regression [13]. The procedure involves the following steps: first, the effect Z-scores for each SNP in both diseases are obtained, and their product is calculated; subsequently, weighted regression of this product is performed against the LD score of the Single-Nucleotide Polymorphism (SNP). The regression slope represents the genetic covariance between the two diseases, which, when combined with SNP heritability estimates for each disease, allows calculation of the global genetic correlation coefficient, which ranges from [−1,^1^]. This method’s advantage lies in its independence from individual genotype data, its insensitivity to sample overlap, and its ability to utilize all SNP signals, rather than being confined to significant loci, thereby comprehensively capturing potential genetic associations between PBC and the four autoimmune diseases (MS, PS, RA, SS).

When using the HDL method to estimate global genetic correlation, GWAS summary data from PBC and target immune diseases (*e.g*., MS, PS, RA, SS) are integrated into a single analytical framework. HDL first utilizes reference genome data to precisely characterize the LD structure among SNPs across the genome, then combines this LD information with each SNP’s effect values in both diseases to construct a statistical model that simultaneously reflects the genetic characteristics of both diseases [14]. By maximizing the model’s fitting performance, HDL is capable of estimating the genetic variance and genetic covariance of both diseases simultaneously, and calculating the genetic correlation coefficient accordingly, thereby reflecting the degree of genetic similarity between the two diseases. Compared to traditional methods, HDL utilizes a more comprehensive set of LD information, thus providing more stable and accurate results, even when sample sizes are limited or correlations between diseases are weak.

#### 2.3.2 Local Genetic Correlation Analyses

In the analysis of local genetic correlations using the LAVA method, the entire genome was initially partitioned into mutually independent blocks based on LD patterns, where SNPs within each block displayed high correlation with one another while remaining largely independent of other blocks [15]. For each genomic region, LAVA integrated GWAS summary statistics from two diseases (*e.g*., PBC and MS, PS, RA, or SS), along with LD information from reference populations, to estimate the genetic effects and similarities between the two diseases within the specific region. The genetic variance for each disease and the genetic covariance between them were computed for each region, which were subsequently utilized to calculate the local genetic correlation coefficient, reflecting the strength of genetic association between the two diseases at that genomic location. Finally, LAVA identified regions exhibiting statistically significant local genetic correlations through statistical testing, thereby facilitating the discovery of “genetic intersections” between PBC and other immune diseases at specific genomic segments, rather than relying solely on overall averages.

#### 2.3.3 CondFDR/conjFDR Analysis

The condFDR/conjFDR analysis for identifying shared genetic loci between two traits begins by calculating the condFDR for each SNP’s *p*-value in one trait, conditional on its significance in another trait, using GWAS summary statistics from both traits [20]. This procedure constructs a bivariate distribution that reorders the association signals of the target trait based on the statistical significance of the other trait, thereby leveraging information from the correlated trait to increase discovery power. Specifically, the condFDR calculation models the joint distribution of SNP p-values across both traits, employing Bayesian methods to estimate the probability that the current trait association represents a false positive, given that the other trait reaches a specified significance level. To identify shared loci between the two traits, the conjFDR method is applied, using the maximum condFDR across the two traits as a joint indicator to screen SNPs that meet the conjFDR threshold (*e.g*., <0.05). This methodology effectively integrates genome-wide information from both traits, significantly enhancing the ability to detect shared genetic signals while controlling the overall false positive rate. Genomic localization analysis of association-significant genetic variants was conducted using the Functional Mapping and Annotation platform [21]. The functional element characteristics of these variant loci and their potential target genes were identified through the SNP2Gene module.

## 3 RESULTS

### 3.1 Global Genetic Correlation

Genome-wide genetic correlation analysis, performed through LDSC methodology, demonstrated statistically significant genetic overlap between PBC and four autoimmune diseases. The most pronounced genetic correlation was identified between PBC and MS (Rg = 0.3427, *p* = 3.00e-04). Furthermore, genetic correlation coefficients were established between PBC and PS (Rg = 0.2346, *p* = 1.00e-04), between PBC and RA (Rg = 0.2249, *p* = 2.00e-04), and between PBC and SS (Rg = 0.2971, *p* = 6.00e-03), with all associations reaching statistical significance (Table **2**).

To further validate the robustness of the aforementioned genetic correlation analysis, HDL was employed as an independent validation approach. The results of the HDL validation analysis were consistent with those of the LDSC primary analysis, thereby confirming, from a methodological standpoint, that stable and reproducible genetic association patterns exist between PBC and the four autoimmune diseases (MS, PS, RA, SS) (Table **2**). This finding provides a robust empirical foundation for elucidating the mechanisms underlying their genetic connections.

### 3.2 Local Genetic Correlation

**Fig. (2):**
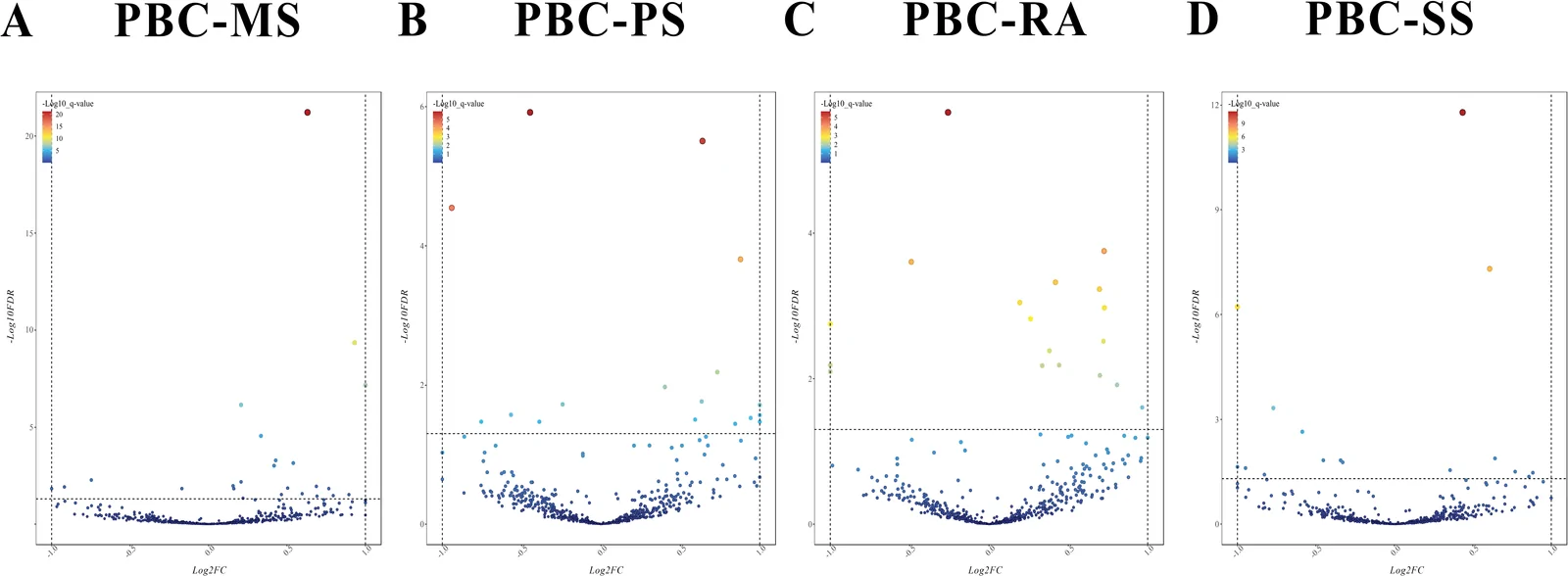
LAVA analysis of PBC and four autoimmune diseases (MS, PS, RA, and SS). The x-axis shows the local genetic correlation, and the y-axis displays the -log10 transformed LAVA values, with a dashed horizontal line indicating significance. Each point represents an SNP, with the border highlighting the lead SNP. The dashed line marks the expected line at a corrected P value of 0.05. (**A**) Genetic association between PBC and MS at the local level. (**B**) Genetic association between PBC and PS at the local level. (**C**) Genetic association between PBC and RA at the local level. (**D**) Genetic association between PBC and SS at the local level. PBC: Primary biliary cholangitis; MS: Multiple sclerosis; PS: Psoriasis; RA: Rheumatoid arthritis; SS: Sjogren's syndrome.

Local genetic correlation analysis revealed intricate genome-wide association patterns between PBC and MS. Based on genome-wide scanning, a total of 17 chromosomal local segments were identified, each meeting statistical significance thresholds for both diseases. These included 6 genomic fragments exhibiting negative genetic correlations, predominantly located on chromosomes 6 and 10, and 11 genomic intervals displaying positive genetic correlations, spanning chromosomes 2-7, 14, 16, and 19 (Fig. **2A**, Supplementary Table **1**).

In the genomic association analysis between PBC and PS, 18 chromosomal intervals with statistically significant associations were identified. Of these, 5 segments displayed negative genetic correlation, concentrated on chromosomes 3 and 6, while the remaining 13 intervals exhibited positive genetic correlation across chromosomes 1, 2, 4, 5, 6, 7, 10, 12, and 19 (Fig. **2B**, Supplementary Table **2**).

Local genomic correlation screening between PBC and RA identified 24 highly significant association regions. Among these, 4 segments demonstrated negative association characteristics, located on chromosomes 6, 7, and 22, while 20 intervals presented positive association patterns, distributed across chromosomes 1, 2, 4, 5, 6, 7, 11, 12, 14, 18, and 19 (Fig. **2C**, Supplementary Table **3**).

Regarding the analysis results for SS, 16 chromosomal local intervals with statistical significance were shared with PBC. A total of 9 negative association segments were identified, primarily located on chromosomes 2, 6, 9, and 12, while 7 positive association segments were found, covering chromosomes 2, 6, 7, 12, 16, and 21 (Fig. **2D**, Supplementary Table **4**).

The systematic genetic association analysis at the chromosomal level described above constructed a complex genomic interaction map between PBC and the four autoimmune diseases, providing an important theoretical framework for further elucidating the genetic basis of shared pathogenic mechanisms among these diseases.

### 3.3 ConjFDR Analysis Identifies Shared Genomic Loci Between Two Traits

**Fig. (3):**
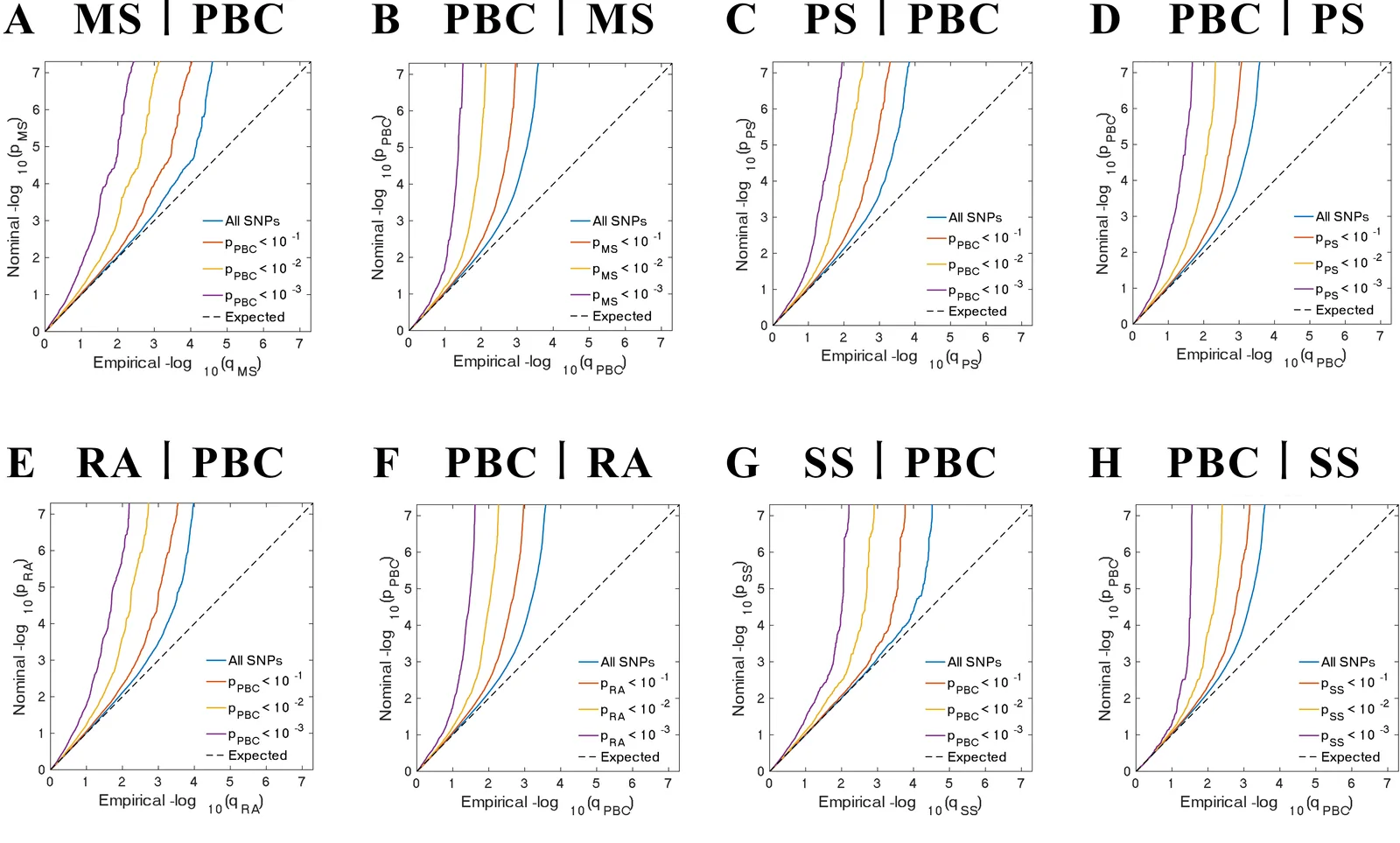
Conditional quantile-quantile plot. The X-axis represents the empirical -log10 of the p-values (or q-values), while the Y-axis shows the nominal -log10 of the p-values (or q-values) associated with the SNPs, indicating the significance of their associations. The conditional Q-Q plot compares the nominal and observed -log10 P values for the primary phenotype against the significance of SNP associations with the secondary phenotype across different P-value thresholds: P < 1.00, P < 0.1, P < 0.01, and P < 0.001. The expected line under the null hypothesis is represented by the dashed line, with any leftward shift indicating the extent of pleiotropic enrichment. (**A**) MS-PBC. (**B**) PBC-MS. (**C**) PS-PBC. (**D**) PBC-PS. (**E**) RA-PBC. (**F**) PBC-RA. (**G**) SS-PBC. (**H**) PBC-SS. PBC: Primary biliary cholangitis; MS: Multiple sclerosis; PS: Psoriasis; RA: Rheumatoid arthritis; SS: Sjogren's syndrome.

Statistical analysis using a Quantile-Quantile (Q-Q) plot demonstrated a strong genetic association between PBC and four autoimmune disorders (MS, PS, RA, SS) (Fig. **3**). The study observed that as the statistical significance for one phenotype increased, the corresponding association values for another phenotype showed a distinct left-skewed distribution. This shift in distribution not only confirmed the genetic relationship between the diseases but also indicated that they may share common genetic loci associated with their pathogenic variants.

Through a detailed analysis of the Q-Q plot, we observed a notable genetic overlap between PBC and the four aforementioned autoimmune diseases. As the genetic association signal for one disease intensified, the statistical distribution of the corresponding disease exhibited a systematic leftward skew. This phenomenon strongly supports the hypothesis of a shared genetic basis for the diseases and suggests that potential shared pathogenic variants may play a crucial role in their pathogenesis.

**Fig. (4):**
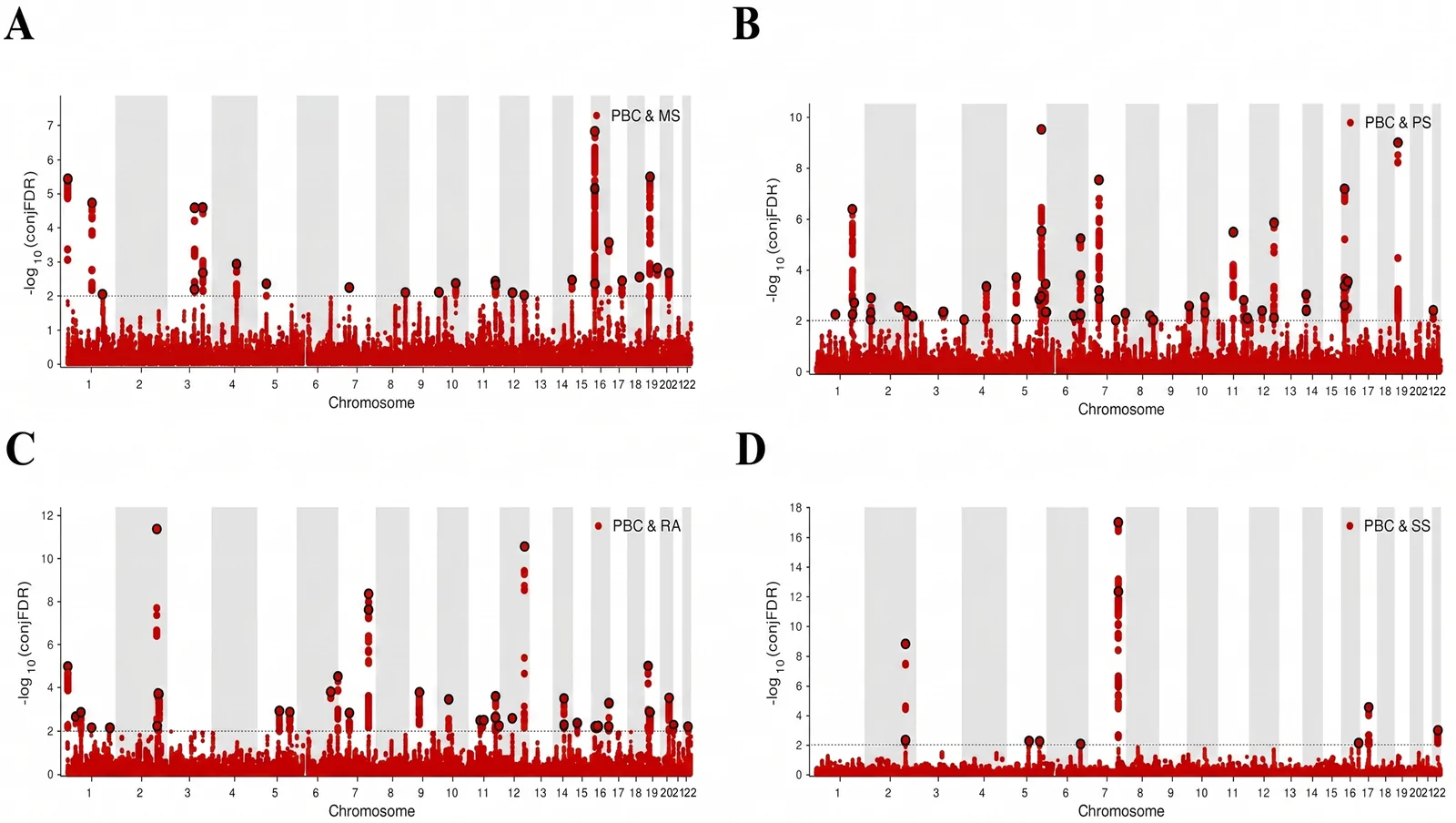
The x-axis displays the chromosomal position, and the y-axis shows the -log10 transformed conjFdr values, with a dashed horizontal line marking significance. Each point represents an SNP, with the border indicating the lead SNP. (**A**) ConjFDR Manhattan plot depicting PBC and MS. (**B**) ConjFDR Manhattan plot depicting PBC and PS. (**C**) ConjFDR Manhattan plot depicting PBC and RA. (**D**) ConjFDR Manhattan plot depicting PBC and SS. The shared risk loci between PBC and the four autoimmune disorders (MS, PS, RA, and SS) have been highlighted. Statistically significant causality is determined when the ConjFDR value is less than 0.01. PBC: Primary biliary cholangitis; MS: Multiple sclerosis; PS: Psoriasis; RA: Rheumatoid arthritis; SS: Sjogren's syndrome.

To investigate the shared genetic basis between PBC and four autoimmune diseases, a systematic assessment was conducted using the conjFDR analysis method. Under the statistical significance threshold of conjFDR < 0.01, 27 genetic loci were identified as commonly involved in PBC and MS, corresponding to 25 functional genes (Fig. **4A**, Supplementary Table **5**). Based on the statistical significance of the min_conjfdr value, lower values of this parameter indicate higher credibility of the corresponding loci in exerting common regulatory effects in both disease phenotypes, while simultaneously reflecting a stronger genetic association between the diseases [20]. In the PBC-MS association analysis, the 10 candidate genes with the lowest min_conjFDR values were identified as: CLEC16A, MAST3, FAM213B, RMI2, CD58, IL12A-AS1, CD86, RP11-542M13.3, MANBA, and SPIB (Supplementary Table **5**).

In the genetic association analysis between PBC and PS, 50 shared genetic loci were detected, encompassing 47 related genes (Fig. **4B**, Supplementary Table **6**). Among these, the top 10 key genes ranked by min_conjfdr values were: AC008697.1, TYK2, ELMO1, RMI2, DENND1B, ATXN2, UBLCP1, CCDC88B, RP11-95M15.2, and TNFAIP3 (Supplementary Table **6**). The genetic association analysis for RA identified 36 shared genetic loci, each mapped to 34 functional genes (Fig. **4C**, Supplementary Table **7**). The top 10 important genes ranked in ascending order of min_conjfdr values included: STAT4, RPL6, IRF5, RP11-128A6.3, TYK2, RP3-395M20.9, CCR6, RP11-95M15.1, FAM205A, and CTLA4 (Supplementary Table **7**). The genetic association analysis between PBC and SS demonstrated that these two conditions shared 10 genetic loci, corresponding to 10 related genes (Fig. **4D**, Supplementary Table **8**), specifically including: IRF5, RP11-128A6.3, STAT4, PLEKHM1, RPL3, NAB1, C5orf30, TNIP1, RP11-542M13.2, and TNFAIP3 (Supplementary Table **8**). These systematic research findings provide important empirical evidence for a more in-depth understanding of the genetic association mechanisms among these autoimmune diseases.

## 4 DISCUSSION

This study integrated several advanced genetic analysis techniques (*e.g*., LDSC, HDL, LAVA, and conjFDR methods) to systematically elucidate the genetic association characteristics between PBC and four autoimmune diseases. The research findings not only validated the genetic correlations between these diseases at the genome-wide level but also precisely identified shared patterns of local genetic effects at chromosome-specific regional levels and successfully identified shared candidate genes that play pivotal regulatory roles. These findings provide novel theoretical insights and scientific evidence for a deeper understanding of the genetic mechanisms underlying comorbidity in these diseases.

Our analyses indicate substantial genetic overlap between PBC and MS. PBC showed a relatively strong genome-wide genetic correlation with MS and shared 17 significant local correlation regions. ConjFDR analysis further identified 27 shared loci (conjFDR < 0.01) mapping to 25 genes, among which CLEC16A, CD58, CD86, and SPIB were prioritized as top candidates. These findings are consistent with previous clinical observations and genetic studies suggesting comorbidity or shared susceptibility between PBC and MS [22, 23], including cross-phenotype GWAS meta-analyses and colocalization-based evidence [24]. Mechanistically, several prioritized genes converge on pathways related to immune tolerance and lymphocyte activation. For example, CLEC16A is implicated in autophagy and immune regulation, and its dysregulation may contribute to autoimmune responses in PBC and neuroinflammatory processes in MS [25, 26]. In addition, CD58 and CD86 are key immunoregulatory molecules involved in T-cell activation and co-stimulation, providing a plausible explanation for the shared breakdown of immune tolerance across both conditions [27-30]. SPIB, a transcription factor associated with B-cell differentiation and memory B-cell programs, may further support persistent immune activation across hepatic and central nervous system compartments [31-33]. Collectively, our multi-layer evidence (genome-wide correlation, locus-specific overlap, and conjFDR-prioritized genes) further refines the shared genetic architecture of PBC and MS and highlights several key candidate genes for functional validation.

Clinical cohorts suggest an association between PBC and PS, with PS reported in 1.5% of 1,554 PBC patients [34] and additional case-control studies providing additional support [35]. Genetic evidence is consistent: Mendelian randomization indicates a positive causal relationship, and polygenic risk scores for PS predict increased PBC risk (top *vs.* bottom quartile: OR = 2.71, 95% CI: 2.13-3.45) [36]. Two shared candidates, TYK2 and TNFAIP3, provide plausible mechanistic links. TYK2 regulates IL-12/IL-23 and type I interferon signaling [37], supporting Th1/IFN-γ-related bile duct injury in PBC [38] and Th17/IL-17/IL-22-driven keratinocyte hyperproliferation in PS [39]. TNFAIP3 (A20), a negative regulator of NF-κB, may promote persistent inflammation when impaired, contributing to NF-κB-dependent inflammatory activation in both biliary epithelium and skin [40, 41].

In a cohort of 1,032 PBC patients and 1,041 controls, Rheumatoid Arthritis (RA) was the most common comorbid autoimmune disease [42]. Genetic evidence supports this link: MR analyses indicate that RA increases PBC susceptibility (OR = 1.28, 95% CI: 1.10-1.40, *p* = 0.001) and further suggest a positive causal relationship between the two conditions [43, 44]. Mechanistically, shared candidates such as STAT4, IRF5, and TNFAIP3 implicate dysregulated immune signaling. STAT4, activated by IL-12/IL-23 and type I interferons, may promote Th1 differentiation and IFN-γ production *via* the JAK-STAT pathway, contributing to bile duct injury in PBC and synovial inflammation in RA [45-47]. IRF5 has been linked to aberrant TLR-MyD88 signaling and altered type I interferon responses, providing an additional shared inflammatory mechanism [38, 48].

Recent evidence supports a strong association between PBC and SS. A meta-analysis of 17 studies reported a pooled SS prevalence of 35% (95% CI: 28-41%, *p* < 0.01) among PBC patients [49]. Bidirectional Mendelian randomization further suggested reciprocal causal effects, with genetically predicted SS increasing PBC risk (OR = 1.737, 95% CI: 1.280-2.357, *p* < 0.001) and PBC also increasing SS risk (OR = 1.174, 95% CI: 1.107-1.246, *p* < 0.001) [50]. Clinically, a retrospective cohort comparing PBC-SS comorbidity with SS alone found higher immunoglobulin levels (IgM, IgG2, IgG3), more pronounced liver enzyme abnormalities, and a higher proportion of very high ANA titers (≥1:10,000) in the comorbidity group [51]. Mechanistically, shared genes such as STAT4 and TNFAIP3 may underlie this overlap. STAT4 can drive Th1/Th17 polarization and IFN-γ/IL-17-related inflammation, contributing to bile duct injury in PBC and glandular inflammation in SS [52-54]. TNFAIP3 (A20), a key negative regulator of inflammatory signaling, has also been implicated in autoimmune overlap and may represent an additional shared susceptibility component [41, 55].

## 5 STUDY LIMITATIONS

Although multiple analytical methods were employed in this study, yielding significant genetic results, several limitations persist. The existing statistical methods are unable to fully eliminate the potential impact of LD between genes on the findings. It is also possible that sample overlap exists between distinct research cohorts, which could compromise the accuracy of the results. Furthermore, GWAS databases lack comprehensive records of lifestyle factors, such as physical activity and alcohol consumption, which may influence the interpretation of genetic associations. This study was based solely on data from European populations, and given the genetic variability across different populations, the applicability of these conclusions to other populations remains to be validated. Future research should be conducted in multi-ethnic populations to confirm the findings of this study. Additionally, the lack of experimental validation of the function of key candidate genes limits our ability to fully understand their mechanisms of action. Subsequent studies should incorporate CRISPR gene editing and other genomic technologies to elucidate the specific biological roles of these genes.

## CONCLUSION

This study, through systematic genomic analysis, provides the first genetic evidence of significant gene overlap between PBC and several autoimmune diseases (MS, PS, RA, SS), offering strong support for their clinical relevance. A set of susceptibility loci, common to both PBC and the mentioned diseases, was identified, and these loci may serve as key targets for future prevention, control, and treatment strategies. These results deepen understanding of the shared pathogenic mechanisms underlying PBC and multiple autoimmune diseases, while also supporting targeted screening of vulnerable populations and the development of personalized intervention approaches that could guide clinical decisions and treatments.

## Figures and Tables

**Fig. (1):**
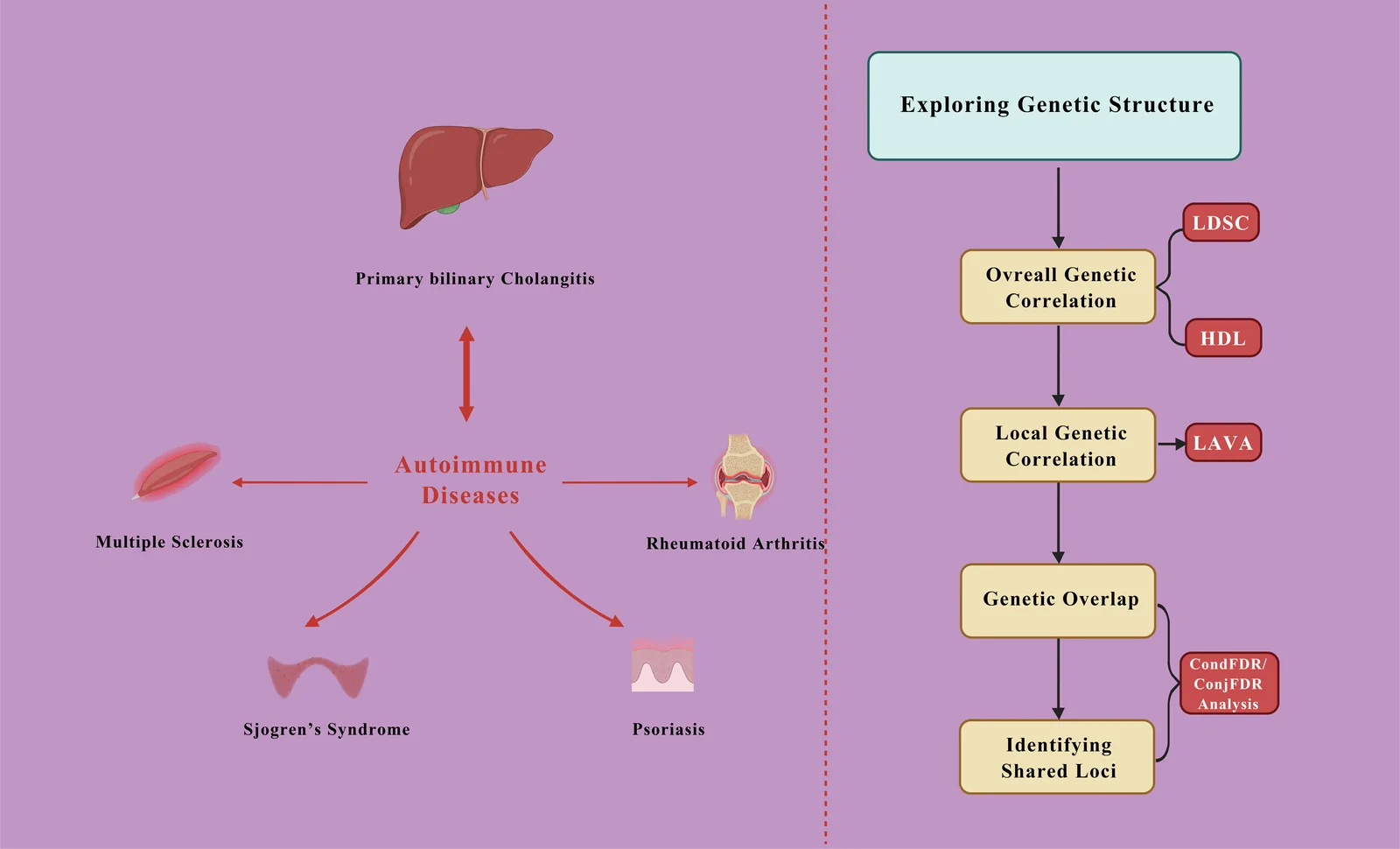
Study flowchart. This figure was created using BioRender. **Abbreviations:** LDSC, Linkage Disequilibrium Score Regression; HDL, High-Dimensional Likelihood; LAVA, local analysis of variant association; cond/conjFDR, conditional and conjunctional false discovery rate.

**Table 1:** Date sources. PBC: Primary biliary cholangitis; MS: Multiple sclerosis; PS: Psoriasis; RA: Rheumatoid arthritis; SS: Sjogren's syndrome.

Phenotypes	Phenotypic Code	Cases/Controls	Ancestry
PBC	ebi-a-GCST90061440	8,021/16,489	European
MS	G6_MS	2,926/495,931	European
PS	L12_PSORIASIS	12,760/482,181	European
RA	M13_RHEUMA	16,314/315,115	European
SS	M13_SJOGREN	3,309/484,260	European

**Table 2:** Overall genetic correlation between PBC and four immune diseases (MS, PS, RA, SS).

Trait1	Trait2	LSDC-Rg	LSDC-P	HDL-Rg	HDL-P
PBC	MS	0.3427	3.00e-04	0.5381	1.36e-06
PBC	PS	0.2346	1.00e-04	0.407	1.30e-04
PBC	RA	0.2249	2.00e-04	0.33	8.69e-06
PBC	SS	0.2971	6.00e-03	0.6717	1.72e-02

**Abbreviations:** PBC: Primary Biliary Cholangitis; MS: Multiple Sclerosis; PS: Psoriasis; RA: Rheumatoid Arthritis; SS, Sjogren's syndrome; LDSC, Linkage Disequilibrium Score Regression; HDL, High-Dimensional Likelihood; Rg, genetic correlation.

## Data Availability

All GWAS data and statistical software used in this study were publicly available (accessible *via* the following URLs), and all results were provided in the main text and supplemental data. IEU database: https://gwas.mrcieu.ac.uk/ FinnGen: https://r12.finngen.fi/ LDSC:https://github.com/bulik/ldsc GNOVA: https://github.com/xtonyjiang/GNOVA HDL: https://github.com/zhenin/HDL LAVA: https://github.com/josefin-werme/LAVA conjFDR:https://github.com/precimed/pleiofdr FUMA:https://fuma.ctglab.nl

## LIST OF ABBREVIATIONS

PBC: Primary Biliary Cholangitis
MS: Multiple Sclerosis
PS: Psoriasis
RA: Rheumatoid Arthritis
SS: Sjögren's Syndrome
GWAS: Genome-Wide Association Studies
LDSC: Linkage Disequilibrium Score Regression
HDL: High-Definition Likelihood
LAVA: Local Analysis of Variant Association
cond/conjFDR: Conditional and Conjunctional False Discovery Rate
SNP: Single Nucleotide Polymorphism
FDR: False Discovery Rate
Q-Q: Quantile-Quantile
conjFDR: Conjunctional False Discovery Rate
Rg: Genetic Correlation
FUMA: Functional Mapping and Annotation of Genetic Associations
